# Positive Mental Well-Being in Children: A Preliminary Structural Validation of the Factor Structure of the Warwick-Edinburgh Mental Wellbeing Scale

**DOI:** 10.3390/children13070941

**Published:** 2026-07-17

**Authors:** Jon Lituri, Stephen Houghton, Jian Zhao

**Affiliations:** Graduate School of Education, The University of Western Australia, Nedlands, Perth, WA 6009, Australia; jlituri@abbc.wa.edu.au (J.L.); jian.zhao@uwa.edu.au (J.Z.)

**Keywords:** positive mental well-being, school screening, children, warwick-edinburgh mental wellbeing scale

## Abstract

**Highlights:**

**What are the main findings?**

- The Warwick-Edinburgh Mental Well-being Scale demonstrated unidimensionality satisfactory model fit, along with satisfactory internal consistency when used with children.
- Invariance testing revealed a satisfactory fit for gender but not school year levels.

**What are the implications of the main findings?**

- The Warwick-Edinburgh Mental Well-being Scale shows potential for school-based screening or monitoring of mental well-being in real-world ecologically valid settings like schools.
- This enhances the potential to generate quality data that identifies trends and determines the effectiveness of interventions to improve children’s mental well-being.

**Abstract:**

Background/Objectives: Promoting positive mental well-being (PMW) among primary school-aged children can reduce future risks of developing adverse mental health. However, there is a dearth of appropriate psychometrically sound measures of PMW that school psychologists, educators, and allied health professionals can utilise with children. The present study sought to provide preliminary evidence pertaining to the suitability of the Warwick-Edinburgh Mental Well-being Scale (WEMWBS) for use with children. Methods: To conduct a preliminary structural validation of the factor structure of the WEMWBS, it was administered to 569 children (328 males, 238 females, 3 did not report their gender) aged 6 to 10 years of age from 14 Western Australian primary schools. Results: An exploratory factor analysis (EFA) from a stratified split-random sample (*n* = 281) yielded a single-factor solution. A confirmatory factor analysis (CFA) on the second half of the sample (*n* = 288) provided support for the hypothesised unidimensional structure of the measure. Multi-group CFA supported configural and metric invariance across gender, but invariance across individual school year levels was not clearly supported. Conclusions: While the findings provide preliminary support for the suitability of the WEMWBS as a measure of children’s PMW in real-world, ecologically valid settings like schools, caution is required for use with younger-aged children.

## 1. Introduction

Worldwide there has been an increasing emphasis on promoting children’s mental well-being within the context of their education [1,2]. This is unsurprising given the continuing evidence that the mental health of children and adolescents is declining [3,4,5], aided in no small part by the (SARS-CoV 2) COVID-19 pandemic and its associated school closures, stay-at-home and social distancing orders [6]. Systematic reviews and meta-analyses show that prevalence estimates of depression and anxiety symptoms are not only significantly higher than prior to the onset of COVID-19 [7,8] but are among the top five causes of overall disease burden. The 2025 second Lancet Commission on adolescent health and well-being [9] argued globally that the health and well-being of 10–24-year-olds is at a tipping point.

The term mental health not only relates to the absence of disorders and deficiencies but also includes a positive aspect, often referred to as positive well-being. These separate but overlapping constructs [10,11] assert a person with a mental disorder may experience a high level of subjective mental well-being, whereas a person without a mental disorder may experience a low level of subjective mental well-being. That is, mental health as a well-being dimension is distinct from, but related to, the mental illness/psychiatric disorder dimension [12] and a person identified as having a mental health difficulty can be flourishing on one hand, but someone without a psychiatric disorder can be languishing. In other words, the absence of psychopathology does not guarantee the presence of well-being [13].

Developing positive mental well-being (PMW) is seen as one way of reducing future risk of psychopathology [13] and the social burden of mental health disorders and its catastrophic outcomes during adolescence and later in life [14]. The evidence is clear that high levels of mental well-being “independently predict less subsequent mental illness and a range of positive effects on individuals and society” [15], p. 2136. However, limited research has focused on primary school-aged children [16], in part due to a distinct lack of appropriate psychometrically sound measures for screening PMW, especially in schools [17].

Researchers have also cited a lack of consensus about terminology and definitions of mental health and well-being [18] and the interchangeability of terms such as PMW, psychological well-being, and mental health in both research and policy as major reasons for the limited research [19]. According to [20], p. 10, the “current body of work is fragmented, with many different conceptualizations and terms being used”. There are, however, other challenges in measuring PMW in children, including, for example, children’s attention span; literacy and comprehension concerns pertaining to self-reports; and the potential for bias and misrepresentation (e.g., children can have the tendency to present themselves in a favourable light, regardless of their true feelings (for a comprehensive review see [21]) especially when these are based on the observations of adults [22,23,24,25].

Nevertheless, reviews have identified a number of potential measures of PMW. For example, ref. [25] identified 22 measures. A range of criticisms were raised about these measures, however, that identified conceptualisation of constructs as problematic; there was a lack of sufficient psychometric evidence for the measures, and development practices for the measures were insufficient. It was also argued that these measures had not integrated hedonic well-being (happiness, feeling good) and eudaemonic well-being (feeling and functioning well), which in combination contribute to a young person being mentally well [12]. As succinctly summarised, there is a “lack of contextually appropriate, usable, and technically sound measures for performing school-based screening” [26], p. 3.

While some of the available measures are more appropriate than others, almost all are for use with adolescents and not specifically designed with children in mind. Furthermore, they do not assess both hedonic and eudaimonic well-being and report limited psychometric testing. Our own searches for child measures of PMW revealed few measures. Examples include the Stirling Children’s Wellbeing Scale [27], a unidimensional 15-item self-report measure of well-being for children aged between 8 and 15 years. It has good construct validity and internal consistency, but lower test–retest reliability when used with younger children (see [28]). The COMPAS-KIDS Wellbeing Scale [29] measures hedonic and eudaimonic mental well-being in children aged 5–12 years. Testing has shown COMPAS-KIDS to be a reliable, stable and valid measure of mental well-being in children aged 5 to 12 years, but borderline to acceptable internal reliability has been found with children aged 5–7 years.

The most widely used measure of PMW is the Warwick-Edinburgh Mental Wellbeing Scale (WEMWBS [30]). The WEMWBS has been adapted across a wide range of countries [31], settings, and samples (adults, adolescents and children, including 6–10-year-olds), with or without neuro-diversities and physical and/or mental health conditions [32,33,34]. Furthermore, it has been administered in schools, and there is satisfactory evidence available about its psychometric properties [35]. This is important because measuring PMW in real-world, ecologically valid settings like schools, where valuable data can be gathered, offers the potential to bridge gaps in students’ needs by fostering positive actions that directly enhance their PMW in context [17].

Schools have been identified as a vital environment for promoting PMW [9,36,37] and mitigating some of the negative impacts of other factors [1,38,39,40]. However, for screening or monitoring PMW in children, appropriate measures are necessary [17], and of the few available, the WEMWBS appears to offer potential. As such, the main purpose of this research was to conduct a preliminary structural validation of the factor structure of the WEMWBS, including model fit, to determine its suitability for use with children aged 6 to 10 years.

## 2. Materials and Methods

### 2.1. Participants and Settings

The total sample comprised 569 children (328 males, 238 females, 3 participants who did not report their gender) aged between 6 and 10 years in the metropolitan area of Perth, the capital city of Western Australia. Of these, 46 were from school year grade 2 (male 30, female 15, one did not report their gender, ages 6–7 years), 54 were from school year 3 (male 33, female 20, one did not report their gender, ages 7–8 years), 386 were from school year 4 (male 219, female 166, one did not report their gender, ages 8–9 years), and 83 were from school year 5 (male 46, female 37, ages 9–10 years).

These children were recruited from 14 randomly selected primary schools, of which 9 were state government schools and 5 were non-government schools. The Index of Community Socio-Educational Advantage (ICSEA) for these schools revealed they were located across a range of socio-economic areas. ICSEA is set at an average of 1000 (SD = 100), and the higher the ICSEA value, the higher the level of educational advantage of students who go to this school and vice versa [41]. The ICSEA values of the 9-state government primary schools ranged from 869 to 1170, while the 5 non-government primary schools ranged from 1049 to 1198.

### 2.2. Instrumentation

The Warwick-Edinburgh Mental Wellbeing Scale [30], developed to assess positive mental well-being (PMW) at a general population level, has been translated into 22 different languages. It is one of the most extensively used and validated scales to measure mental well-being in clinical and community settings because of its high acceptability, having items related to both eudaimonic and hedonic aspects of well-being along with its use as a reliable measure to evaluate interventions [42]. There is also a brief seven-item version (Short Warwick-Edinburgh Positive Mental Wellbeing Scale: SWEMWBS), which was developed using the Rasch measurement model [43,44]. The SWEMWBS performs similarly to the full WEMWBS and is useful where large batteries of questionnaires are being administered [45], which was not the case in the present study. Overall, an advantage of the WEMWBS over similar scales is that it assesses well-being over a wide range of feelings, including one’s own feelings towards self and towards others, and its positive nature makes it highly acceptable to respondents [42].

In the WEMWBS, participants respond to 14 positively worded items using a five-point Likert scale (scored 1 “none of the time”, 2 “rarely”, 3 “some of the time”, 4 “often”, 5 “all of the time”). Once completed, a total score of 14 to 70 is possible. Responses are based on the participants’ feelings over the previous two weeks, with higher scores indicating higher levels of PMW. Examples of items include “I’ve been feeling cheerful”, “I’ve been feeling optimistic about the future”, “I’ve been thinking clearly” (for a full description of the WEMWBS development, see [30]).

Research conducted with Australian 8- and 9-year-old children [34] and 13- to 16-year-old adolescents [35] has highlighted potential difficulties with the item phrased ‘feeling optimistic’. Therefore, as adopted by [34] in a study involving children, the word “positive” was inserted in parentheses after the word “optimistic” in the present research.

The readability levels of the WEMWBS were assessed prior to its use with children in the present research using the Flesch-Kincaid Grade Level and the Flesch Reading Ease tests [46]. In this present study, the Flesch-Kincaid Grade Level (3.9) and the Flesch Reading Ease (76.2) scores for the WEMWBS were equivalent to Australian Grades 4–5 (approximately 8–10 years of age). The Flesch Reading Ease score (76.2) represented the easier end of “fairly easy”. Eight words in the WEMWBS were found to be relatively complex, which represented 7% of the total words in the instrument. Therefore, it was decided that prior to beginning the WEMWBS administration in the present study, teachers of Grades 2 and 3 children would provide a brief explanation about the response options and would read the WEMWBS items verbatim so that the children could follow them and then complete each item.

### 2.3. Procedure

The Human Research Ethics Committees of the administering institution (RA/4/20/6117) and the Western Australian Department of Education (RA/4/20/1039) and the principals of the non-government schools granted permission to conduct this research. Initially, 20 schools were randomly identified across the four areas of the Perth metropolitan area. The principals of these schools were approached and provided with an explanation of the research. Of the 20, 14 agreed to be involved, and information sheets and consent forms were sent to the parents of children in school year levels 2 to 5 (6–10 years of age) at these schools. Only children for whom informed consent was obtained were included in the study.

To ensure the WEMWBS was administered consistently across schools, one teacher in each school liaised with the researchers and administered the survey following standardised instructions. Children completed the WEMWBS in groups of 6–25 during regular class time via an online survey using Qualtrics. Each participant received a unique identification code to log on to the survey to ensure confidentiality. On average, the WEMWBS took approximately 10–15 min to complete.

### 2.4. Statistical Analysis

Data were analysed using a combination of IBM SPSS Version 27 [47] and LISREL Version 11 [48] for exploratory and confirmatory factor analyses, respectively. LISREL 11 [48] is widely applied in the social and behavioural sciences to analyse complex relationships between observed and unobserved (latent) variables. Prior to conducting any analyses, data screening tests for all the relevant assumptions of each intended statistical procedure were performed to ensure compliance with these underlying assumptions.

Five indices were utilised to assess the goodness of fit of the model: the comparative fit index (CFI) and non-normed fit index (TLI/NNFI) (CFI and TLI: above 0.95 indicates good fit, above 0.90 indicates adequate fit), the root mean-square error of approximation (RMSEA: 0.05 or less indicates good fit, 0.08 or less indicates adequate fit), and chi-square (non-significant values represent good fit). The routine use of the RMSEA is strongly recommended [49] as (i) it is adequately sensitive to model misspecification, (ii) its commonly used interpretive guidelines appear to yield appropriate conclusions regarding model quality, and (iii) it is possible to build confidence intervals around RMSEA values (see [50]).

## 3. Results

An exploratory factor analysis (EFA) and a confirmatory factor analysis (CFA) were conducted. A stratified random split sample with approximately equal distribution of children by gender from each school grade level was generated. The EFA was conducted on one-half of the sample (*n* = 281). A CFA was fit to the second half (*n* = 288) to confirm the model fit of the factor structure identified in the EFA. The data were then used to test the measurement invariance of the factor structure across gender and school year level.

### 3.1. Exploratory Factor Analysis of the WEMWBS

The inter-correlations of the 14 items comprising the WEMWBS revealed the presence of a substantial number of correlation coefficients above 0.3, indicating underlying relationships (see Table 1). Bartlett’s test of sphericity [51], *χ*^2^(91) = 1293.32, *p* < 0.001 and Kaiser–Meyer–Olkin’s [52,53] measure of sampling adequacy (MSA) of 0.91 supported the factorability of the correlation matrix.

The means and standard deviations of the 14 WEMWBS items are presented in Table 2.

An unrestricted maximum likelihood (ML) factor analysis using IBM SPSS version 27 [47] revealed the presence of two possible factors with eigenvalues exceeding 1.0, explaining 39.35% and 8.94% of the variance, respectively. A visual examination of the scree plot suggested a probable one-factor solution. The factor-loading matrix revealed only one item (“Interested in other people”) comprised the second factor. Therefore, the factor analysis was re-run with one factor specified, and this revealed a one-factor solution, explaining 35.03% of the variance with all variables having loadings above 0.30 (see Table 3).

Horn’s parallel analysis [54] with 50 to 1000 replications indicated that for one factor, the eigenvalue from the sample in this study (5.509) exceeded the corresponding eigenvalues (1.36 to 1.38 on various numbers of replications) obtained from the randomly generated data set of the same size. This further suggests that the WEMWBS items were assessing one underlying dimension. The cumulative percentage of variance for the one-factor solution of the 14 items accounted for 35.03%, which is above the suggested minimum accepted level of 30% [55]. Cronbach’s alpha calculated using the EFA split sample was 0.87. These results provided confidence in evaluating the fit of the factor structure using CFA via LISREL 11 with the second half of the data set (*n* = 288).

### 3.2. Confirmatory Factor Analysis of the WEMWBS

To cross-validate the factor structure of the WEMWBS, a confirmatory factor analysis (CFA) was conducted using the CFA split sample (*n* = 283). The CFA split sample initially comprised 288 participants. After listwise deletion of cases with missing responses on one or more WEMWBS items, the CFA was conducted using 283 complete cases. A hypothesised single-factor model was tested, with all 14 WEMWBS items loading onto one latent well-being factor.

**Figure 1:**
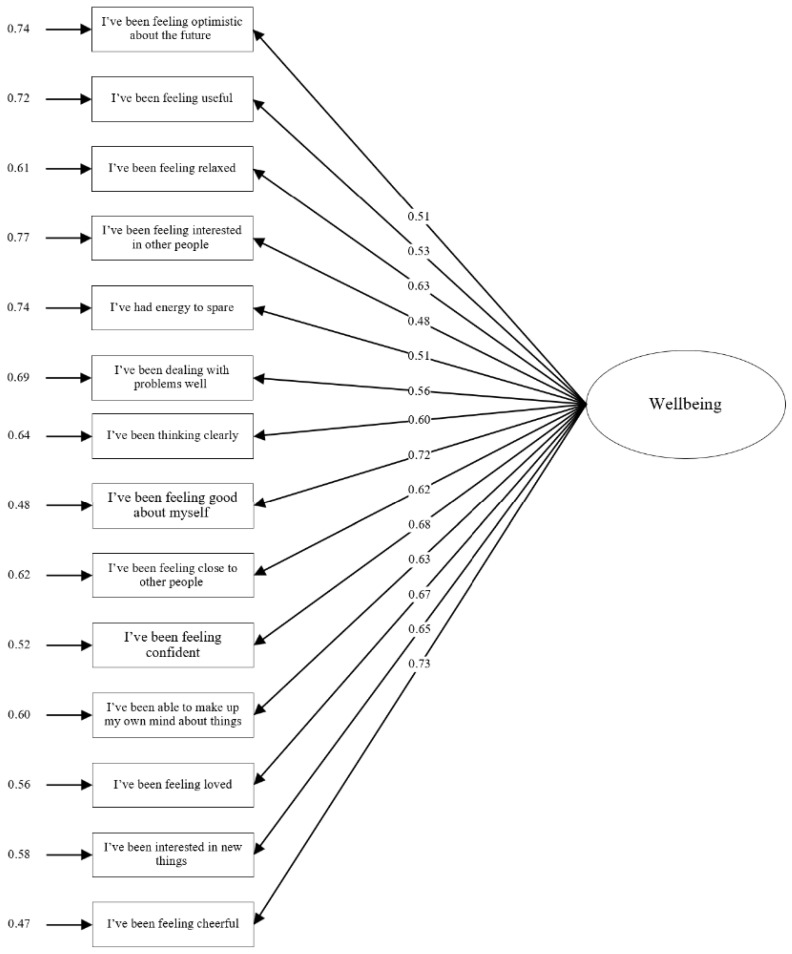
Standardised one-factor confirmatory factor analysis model of the 14-item WEMWBS.

The model (see Figure 1) demonstrated statistically significant item loadings for all 14 items (*p* < 0.001). Standardised factor loadings ranged from 0.48 to 0.73, indicating that most items loaded moderately to strongly onto the latent well-being factor. The strongest loading was observed for Cheerful and Feeling Good (both λ = 0.73), followed by Confident (λ = 0.68), Loved (λ = 0.67), Interested in New Things (λ = 0.65), Make Up My Mind (λ = 0.63), and Relaxed (λ = 0.63). The lowest loading was observed for Interested in Other People (λ = 0.48). The squared multiple correlations (R^2^) ranged from 0.23 to 0.53, suggesting that the latent well-being factor explained between 23% and 53% of the variance in individual items.

Model fit indices indicated an acceptable fit between the single-factor model and the data: *χ*^2^(77) = 170.72, *p* < 0.001; CFI = 0.930; NNFI = 0.917; GFI = 0.919; SRMR = 0.048; and RMSEA = 0.066, 90% CI [=0.052, 0.079]. Although the chi-square test was statistically significant, which is common in larger samples, the approximate fit indices generally supported the adequacy of the one-factor model.

McDonald’s omega [56] was calculated using the standardised factor loadings and error variances obtained from the CFA. The 14-item WEMWBS demonstrated good internal consistency, with McDonald’s omega = 0.89.

Gender measurement invariance was examined using multi-group CFA across male and female participants. The configural model demonstrated an acceptable-to-good fit, χ^2^(154) = 279.45, *p* < 0.001, CFI = 0.964, TLI/NNFI = 0.957, RMSEA = 0.076, 90% CI [0.062, 0.091], supporting the same one-factor structure across gender. Metric invariance was then tested by constraining the factor loadings to equality across males and females. The metric model also demonstrated acceptable-to-good fit, χ^2^(168) = 290.45, *p* < 0.001, CFI = 0.964, TLI/NNFI = 0.962, RMSEA = 0.072, 90% CI [0.058, 0.086]. Model fit did not deteriorate relative to the configural model, ΔCFI = 0.000 and ΔRMSEA = −0.004. These results supported metric invariance across gender, indicating that the WEMWBS items were similarly related to the latent well-being factor for male and female participants.

The configural model across school year levels did not demonstrate adequate fit. Although the model converged, fit indices suggested poor fit: χ^2^(308) = 515.09, *p* < 0.001, CFI = 0.945, TLI/NNFI = 0.935, RMSEA = 0.098, 90% CI [0.083, 0.113]. Inspection of group-specific estimates indicated instability in the Year 2 group, which had a small sample size (*n* = 24) and several negative or non-significant factor loadings. Given that configural invariance was not clearly supported, further invariance testing across school year levels was not pursued.

An independent-samples *t*-test indicated no significant difference in WEMWBS total scores between males (M = 51.68, SD = 9.64) and females (M = 50.26, SD = 10.13), *t*(553) = 1.67, *p* = 0.095. A one-way ANOVA to compare WEMWBS scores across school year levels revealed similar mean scores: Year 2 (M = 52.30, SD = 6.26), Year 3 (M = 52.04, SD = 9.73), Year 4 (M = 50.65, SD = 10.37), and Year 5 (M = 51.96, SD = 9.04). The overall ANOVA was not statistically significant, F(3, 554) = 0.85, *p* = 0.469. Games–Howell post hoc comparisons also indicated no significant pairwise differences between year levels.

Overall, the CFA results provided support for the unidimensional structure of the 14-item WEMWBS in this sample. However, the findings should be interpreted as evidence of acceptable rather than excellent model fit, especially given the weaker support for invariance across school year levels of children.

## 4. Discussion

To increase the mental health of a population requires well-validated measures that provide researchers with the opportunity to investigate, understand and determine what works, for whom, how, and under what circumstances [57]. Valid and reliable measures of children’s PMW are necessary because mental health is more than the absence of illness; it is also about the positive aspects of feeling good (hedonia) and functioning well (eudaimonia) [58]. Research examining PMW in children is limited, however, and robust screening measures for use in schools are scarce.

The measure used in this present research, the WEMWBS, is one of the few instruments that integrates the key elements that, in combination, comprise the broad concept of mental well-being [12,59]. Although it is one of the most widely used measures of PMW with adolescents and adults, its application with children is scarce.

In the present study the WEMWBS demonstrated satisfactory structural validity and internal consistency with 6- to 10-year-old children. Factorial validity was shown through a number of accepted criteria for fit indexes: CFI = 0.930, NNFI = 0.917, GFI = 0.919, SRMR = 0.048, and RMSEA = 0.066, 90% CI [=0.052, 0.079]. While these indices are encouraging, two items in the EFA (but not in the CFA) had the lowest loadings, namely “I’ve been feeling interested in other people” (0.33) and “I’ve had energy to spare” (0.38). Although this was not the case in the CFA, consideration must be given regarding possible comprehension and/or item developmental appropriateness issues. The readability levels required for the WEMWBS were equivalent to Australian Grades 4–5 (approximately 8–10 years of age), and it is likely that some of the younger children (aged 6 to 7 years) experienced difficulties.

Understanding, contextualising, and decontextualising the meanings of words is an ongoing developmental process in children, which becomes more challenging as vocabulary words increase in difficulty [60]. Moreover, irrespective of the modality in which a text is presented to children (e.g., written or read aloud by teachers), to successfully comprehend it requires the construction of an integrated representation of the overall meaning of the text [61]. Many children are competent readers but do not have knowledge of the meanings of the words in the text, and this is something reading comprehension depends on [62]. Therefore, the younger children in this present study may have experienced difficulties imposing meaning to the WEMWBS items “I’ve been feeling interested in other people” and “I’ve had energy to spare”. Moreover, the ambiguity, especially in the first item (i.e., who is referred to by ‘other people’), might cause confusion in children, because they would respond differently to family, close friends and others in general, and hence there is a proneness to misinterpretation.

With reference to model fit, there is little research with children with which to compare the present findings. Unidimensionality, satisfactory model fit, and satisfactory internal consistency, such as found in the present research with children, have frequently been demonstrated in studies using the shorter seven-item version of the WEMWBS with adolescents (i.e., the SWEMWBS), including, for example, with Danish 10–16-year-olds [57], Swedish 14–15-year-olds [63], Polish 14–18-year-olds [64], Czech 15–18-year-olds [65], and Welsh 11–16-year-olds [43,66]. Recent data from N = 132,828 Finnish 13- to 20-year-olds reported a single-factor model with good internal consistency (*ω*  =  0.96) [67]. Investigations of the full 14-item WEMWBS with children and adolescents are fewer in comparison, however. A single underlying PMW construct was found by [59], while other research [35] has revealed model fit, which, while acceptable, was not totally supportive of a single underlying PMW construct. One study [45] administered both the short and long forms of the WEMWBS to Scottish and Northern Irish adolescents and reported both to be psychometrically valid, internally consistent, factor-saturated, and measurement invariant.

With specific reference to children, limited evidence exists from studies using the WEMWBS. Similar to the present study, ref. [34] reported a single underlying PMW construct with acceptable model fit. However, the sample comprised only males. Other measures of PMW have been administered to secondary school adolescents and primary school children along with the WEMWBS within the same cohort. For example, ref. [68] used the WEMWBS with 7570 secondary school adolescents and the Stirling Children’s Wellbeing Scale with 1413 primary school-aged children in the same study. However, it appears that no model fit statistics were reported for the WEMWBS, and similarly, the psychometric properties of the Stirling Children’s Wellbeing Scale were not examined.

In the present study, no differences were found in WEMWBS scores for males and females, which is contrary to some studies using the WEMWBS with adolescents. For example, ref. [69] reported females scored lower than males on the 14-item WEMWBS, as did [70] using the seven-item SWEMWBS. The study by [70] was a longitudinal study with 8612 adolescents that found at ages 11–12 years, females had significantly lower levels of PMW than males and that this deteriorated further year on year. Configural invariance was not fully supported across school year levels in the present study, however, meaning caution should be exercised when interpretating differences between age groups.

## 5. Limitations and Future Research

There are limitations that must be acknowledged. First and foremost, it must be reiterated that this study is a preliminary structural validation of the WEMWBS. There were no tests for convergent validity (e.g., correlation with another well-being or related construct), discriminant validity (e.g., distinction from symptoms or distress), criterion validity (e.g., association with known indicators or outcomes), and test–retest reliability. Future studies should address this now that preliminary evidence of the WEMWBS factor structure shows it is suitable for use with children.

It must also be acknowledged that the samples were obtained from Western Australia, and as such, the generalisability of the findings to children from diverse cultural or socioeconomic backgrounds in other states of Australia or other countries internationally must be borne in mind. Relatedly, the sample was unevenly distributed across school year levels, with Grade 4 substantially overrepresented, which also impacts the generalisability of the findings to the full 6–10-year-old age range.

Social desirability (i.e., the tendency to present oneself in a positive light by over-reporting positive behaviours and under-reporting negative behaviours) especially when self-reporting, must also be taken into consideration as a limitation. As reference [71] asserted, children as young as 7 years are capable of introspection and reporting on their thoughts and feelings, and although self-report may introduce social desirability bias, it is necessary for accessing the subjective experiences of PMW because parents and teachers have difficulty perceiving the internal world of their children. In addition, the class teachers of the younger Grade 2 and 3 children read the WEMWBS items aloud, and while this was a reasonable accommodation, it was a different administrative procedure (compared to that of the older children) and thus may have affected responses. There is evidence that one-on-one delivery of questions with young children elicits significantly more socially desirable answers than classroom delivery [72]. This might also impact how school grade level differences are interpreted.

In conclusion, school-based monitoring or screening is now widely accepted as the first step in designing and providing mental health services for young people [26,41]. Measuring PMW in real-world ecologically valid settings like schools, where valuable data can be gathered, offers the potential to bridge gaps in students’ needs by fostering positive actions that directly enhance their PMW in context [17]. For this to happen, relevant reliable measures of PMW are necessary to generate quality data that identifies trends, determines the effectiveness of interventions to improve children’s mental well-being and reveals emerging challenges [73]. While the present study provides school psychologists, teachers, and student services personnel with preliminary evidence pertaining to the suitability of the WEMWBS as a possible screening or monitoring measure in primary school-aged children, further evidence pertaining to the validity, test–retest reliability, sensitivity and specificity, and responsiveness to intervention of the WEMWBS is required.

## Figures and Tables

**Table 1:** Inter-correlations of the 14 WEMWBS items.

	1	2	3	4	5	6	7	8	9	10	11	12	13	14
1-I’ve been feeling optimistic about the future	1													
2-I’ve been feeling useful	0.41 **	1												
3-I’ve been feeling relaxed	0.26 **	0.34 **	1											
4-I’ve been feeling interested in other people	0.34 **	0.31 **	0.18 **	1										
5-I’ve had energy to spare	0.22 **	0.24 **	0.29 **	0.19 *	1									
6-I’ve been dealing with problems well	0.22 **	0.25 **	0.28 **	0.11	0.13 *	1								
7-I’ve been thinking clearly	0.28 **	0.36 **	0.38 **	0.19 **	0.28 **	0.47 **	1							
8-I’ve been feeling good about myself	0.39 **	0.36 **	0.39 **	0.21 **	0.26 **	0.40 **	0.50 **	1						
9-I’ve been feeling close to other people	0.35 **	0.32 **	0.19 **	0.42 **	0.15 *	0.31 **	0.38 **	0.40 **	1					
10-I’ve been feeling confident	0.37 **	0.37 **	0.35 **	0.16 **	0.29 **	0.39 **	0.53 **	0.60 **	0.38 **	1				
11-I’ve been able to make up my own mind about things	0.37 **	0.26 **	0.30 **	0.14 *	0.26 **	0.48 **	0.47 **	0.49 **	0.22 **	0.47 **	1			
12-I’ve been feeling loved	0.33 **	0.30 **	0.28 **	0.19 **	0.17 **	0.29 **	0.43 **	0.47 **	0.43 **	0.37 **	0.28 **	1		
13-I’ve been interested in new things	0.31 **	0.27 **	0.24 **	0.28 **	0.24 **	0.32 **	0.47 **	0.39 **	0.24 **	0.41 **	0.47 **	0.27 **	1	
14-I’ve been feeling cheerful	0.41 **	0.40 **	0.38 **	0.26 **	0.26 **	0.43 **	0.57 **	0.59 **	0.40 **	0.57 **	0.50 **	0.42 **	0.47 **	1

Note. **. Correlation *p* < 0.01 level (2-tailed). *. Correlation *p* < 0.05 level (2-tailed).

**Table 2:** Descriptive statistics for the 14 WEMWBS items.

Item	*n*	M	SD
I’ve been feeling optimistic about the future	281	3.46	1.17
I’ve been feeling useful	281	3.32	1.13
I’ve been feeling relaxed	280	3.59	1.04
I’ve been feeling interested in other people	278	3.08	1.24
I’ve had energy to spare	280	3.58	1.24
I’ve been dealing with problems well	281	3.38	1.10
I’ve been thinking clearly	281	3.74	1.04
I’ve been feeling good about myself	281	3.96	1.04
I’ve been feeling close to other people	281	3.48	1.18
I’ve been feeling confident	281	3.87	1.01
I’ve been able to make up my own mind about things	281	3.72	1.04
I’ve been feeling loved	281	4.08	1.16
I’ve been interested in new things	281	4.00	1.08
I’ve been feeling cheerful	280	3.90	1.03

Note. Warwick-Edinburgh Mental Wellbeing Scale items are rated on a 1–5 scale, with higher scores indicating higher PMW.

**Table 3:** Factor loadings for the one-factor EFA solution of the 14-item WEMWBS.

Item	Factor Loading
I’ve been feeling cheerful	0.77
I’ve been feeling good about myself	0.75
I’ve been feeling confident	0.73
I’ve been thinking clearly	0.73
I’ve been able to make up my own mind about things	0.63
I’ve been interested in new things	0.59
I’ve been feeling loved	0.56
I’ve been dealing with problems well	0.56
I’ve been feeling optimistic about the future	0.53
I’ve been feeling close to other people	0.53
I’ve been feeling useful	0.52
I’ve been feeling relaxed	0.50
I’ve had energy to spare	0.38
I’ve been feeling interested in other people	0.33

## Data Availability

The research data is available on request from the second author. It is not freely available via a public data repository because of the requirements of the Department of Education.
